# A dynamic nomogram for predicting pathologic complete response to neoadjuvant chemotherapy in locally advanced rectal cancer

**DOI:** 10.1002/cam4.7251

**Published:** 2024-05-31

**Authors:** Guancong Wang, Jiasen Li, Ying Huang, Yincong Guo

**Affiliations:** ^1^ Department of Colorectal and Anal Surgery Zhangzhou Affiliated Hospital of Fujian Medical University Zhangzhou China; ^2^ Department of Interventional Radiology ZhangZhou Affiliated Hospital of Fujian Medical University Zhangzhou China; ^3^ Department of Colorectal Surgery Fujian Medical University Union Hospital Fuzhou China

**Keywords:** dynamic nomogram, locally advanced rectal cancer, neoadjuvant chemoradiotherapy, pathological complete response, total mesorectal excision

## Abstract

**Aim:**

To explore the clinical factors associated with pathologic complete response (pCR) for locally advanced rectal cancer (LARC) patients treated with neoadjuvant chemoradiotherapy (nCRT) and develop a web‐based dynamic nomogram.

**Methods:**

Retrospective analysis of patients with examination confirmed LARC from 2011 to 2022. Patients from the Union Hospital of Fujian Medical University were included as the training cohort (*n* = 1579) and Zhangzhou Hospital of Fujian Medical University as the external validation cohort (*n* = 246).

**Results:**

In the training cohort, after nCRT, 350 (22.2%) patients achieved pCR. More stomas were avoided in pCR patients (73.9% vs. 69.7%, *p* = 0.043). After a median follow‐up time of 47.7 months (IQR 2–145) shown OS (5‐year: 93.7% vs. 81.0%, HR = 0.310, 95%CI: 0.189–0.510, *p* < 0.001) and DFS (5‐year: 91.2% vs. 75.0%, HR = 0.204, 95%CI: 0.216–0.484, *p* < 0.001) were significantly better among patients with pCR than non‐pCR. Multivariable Logistic analysis shown pCR was significantly associated with Pre‐CRT CEA (HR = 0.944, 95%CI: 0.921–0.968; *p* < 0.001), histopathology (HR = 4.608, 95%CI: 2.625–8.089; *p* < 0.001), Pre‐CRT T stage (HR = 0.793, 95%CI: 0.634–0.993; *p* = 0.043), Pre‐CRT N stage (HR = 0.727, 95%CI: 0.606–0.873; *p* = 0.001), Pre‐CRT MRI EMVI (HR = 0.352, 95%CI: 0.262–0.473; *p* < 0.001), total neoadjuvant therapy (HR = 2.264, 95%CI: 1.280–4.004; *p* = 0.005). Meanwhile, the online version of the nomogram established in this study was publicized on an open‐access website (URL: https://pcrpredict.shinyapps.io/LARC2/). The model predicted accuracy with a C‐index of 0.73 (95% CI: 0.70–0.75), with an average C‐index of 0.73 for the internal cross validation and 0.78 (95% CI: 0.72–0.83) for the external validation cohort, showing excellent model accuracy. Delong test results showed the model has an important gain value for clinical characteristics to predict pCR in rectal cancer.

**Conclusions:**

Patients with pCR had a better prognosis, including OS and DFS, and were independently associated with Pre‐CRT CEA, histopathology, Pre‐CRT T/N stage, Pre‐CRT MRI EMVI, and TNT. A web‐based dynamic nomogram was successfully established for clinical use at any time.

## 1 INTRODUCTION

Presently, the standard of treatment for locally advanced rectal cancer (LARC) continues to recommend radical resection after neoadjuvant chemoradiotherapy (nCRT).^1^ It is generally recognized that pathologic complete response (pCR) is associated with better overall survival (OS) (HR = 0.50; *p* < 0.001) and disease‐free survival (DFS) (HR = 0.49; *p* < 0.001) in patients with digestive cancer compared to non‐pCR.^2^ In addition, for patients with pCR, a “watch and wait” or local excision approach appears to be feasible and without loss of oncologic safety.^3^, ^4^ However, response to standard treatment is inconsistent among different LARC patients and pCR has been reported in 13.0%–20.0% of patients after nCRT.^5^, ^6^

To date, multiple large studies aimed to improve the probability of pCR, such as total neoadjuvant therapy (TNT), radiation combined with mFOLFOX6, and prolonged waiting time after radiotherapy.^5^, ^7^, ^8^ However, there may be some correlation between pCR and clinical factors preceding the patient's treatment, and unfortunately, the influences on the achievement of pCR after nCRT have not been completely established. For example, studies have suggested that pCR may be associated with insurance status and time between radiation and surgery,^5^ pretreatment tumor thickness,^9^ as well as distance to the anal verge.^6^

Therefore, it is important to identify the clinical factors associated with obtaining a pCR after nCRT. This study aimed to address the factors associated with obtaining a pCR in LARC patients treated with nCRT and develop a web‐based dynamic nomogram for predicting pCR before making treatment decisions.

## 2 MATERIALS AND METHODS

### 2.1 Patients

We retrospectively analyzed 1825 patients with examination confirmed LARC from 2011 to 2022 at the Union Hospital of Fujian Medical University (Fuzhou, China; included as training cohort) and the Zhangzhou Hospital of Fujian Medical University (Zhangzhou, China; included as external validation cohort).

**FIGURE 1:**
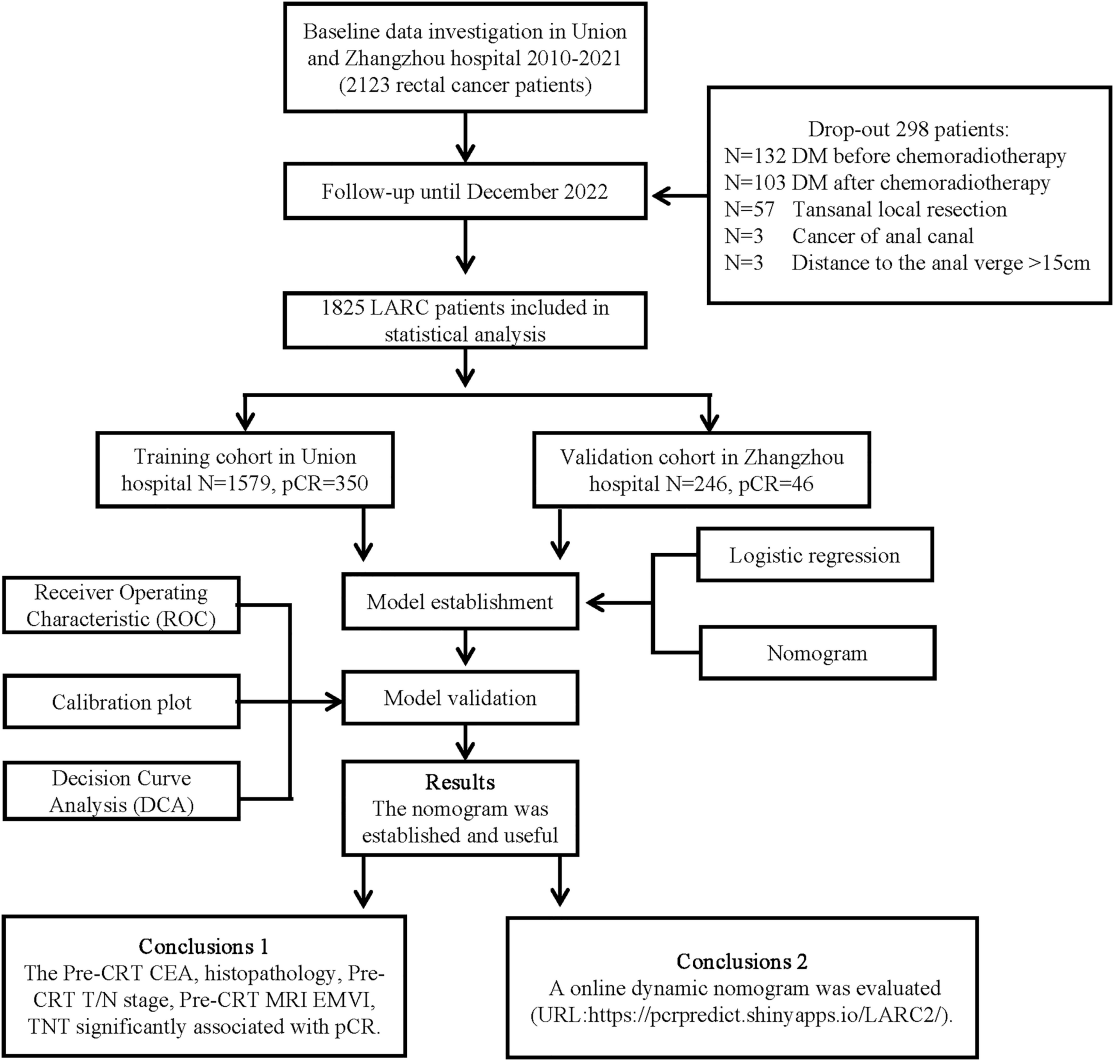
Study flow. LARC, locally advanced rectal cancer; pCR, pathologic complete response.

Inclusion criteria: (1) Patients with a pathologically confirmed diagnosis of rectal cancer; (2) clinical stage of cII or cIII (cT3–4 N0–2 M0) (Based on AJCC staging system, phase 8); (3) distance to the anal verge ≤15 cm; (4) received nCRT combined with total mesorectal excision (TME) and postoperative adjuvant chemotherapy if necessary; (5) complete clinicopathological features, imaging, follow‐up, and clinical data. Patients with distant metastasis before or after nCRT and other malignant diseases were excluded. The Medical Ethics Committee of Zhangzhou Affiliated Hospital of Fujian Medical University (Grant No. 2023LWB141) and Union Hospital of Fujian Medical University (Grant No. 2022kY134) approved this study. (Figure 1).

### 2.2 Tumor response evaluation

Pathologic results were evaluated by two independent pathologists. The pCR was defined as no found tumor cells, complete tumor regression, and only fibroblasts remaining in the resected tumor tissue and regional lymph nodes. (i.e., ypT0N0).^10^

### 2.3 Treatments

All enrolled patients underwent nCRT with either intensity‐modulated radiotherapy (IMRT) or three‐dimensional conformal radiotherapy (3DCRT) in the form of radiotherapy and oral capecitabine during the same period of radiotherapy. The dose of short‐course radiotherapy was 25 Gy/5 times, and the dose of long‐course radiotherapy was 45.0–50.4 Gy/25–28 times. Patients received neoadjuvant chemotherapy at the end of radiotherapy. Neoadjuvant chemotherapy regimen: mFolFox6 (calcium folinate 400 mg/m^2^, fluorouracil 2600 mg/m^2^, oxaliplatin 85 mg/m^2^) or Xelox (oxaliplatin 130 mg/m^2^, capecitabine 1000 mg/m^2^ bid). TME procedure was performed after nCRT.

### 2.4 Follow‐up

Each patient was followed up at 3‐month intervals for the first 3 years, at 6‐month intervals for the next 2 years, and annually thereafter. Follow‐up until December 2022.

### 2.5 Statistical analyses, model development, and validation

The endpoint of building the dynamic nomogram was pCR. Cases were allocated to the training cohort (Union Hospital, *n* = 1579) and external validation cohort (Zhangzhou Hospital, *n* = 246). Differences between cohorts were analyzed using chi‐squared and *t*‐tests. Logistic analysis was used to determine independent predictors for pCR. *p* < 0.05 was considered statistically significant. According to the multifactor regression results, the nomogram was developed and internally cross‐validated in the training cohort and validated in the external validation cohort. DynNomapp was created as a web‐based dynamic and uploaded to Shinyapp internet. The evaluation of the model was based on a consistency index (C‐index) to determine accuracy, a decision curve analysis (DCA) to assess clinical benefit, and a calibration curve to assess the consistency of the model's predicted results with the actual results. The Delong test was used to compare the area under the curve (AUC). The Hosmer‐Lemeshow‐test was used for the test of goodness of fit. SPSS version 26.0 (IBM) and R 4.1.1 (http://www.r‐project.org) software were used for statistical analysis.

## 3 RESULTS

### 3.1 Patient characteristics

The training cohort consisted of 1579 patients who had combined nCRT with TME surgery, and the external validation cohort consisted of 246 patients. The clinicopathologic characteristics of the two groups are shown in Table 1. After nCRT, 350 (22.2%) patients achieved pCR in the training cohort, while 46 (18.7%) achieved pCR in the external validation cohort, and the difference between the two groups was not statistically significant (*p* > 0.05) (Table 1).

**TABLE 1:** General clinical information of patients with locally advanced rectal cancer in the training and external validation cohorts.

Variables	Training cohort (*n* = 1579)	Validation cohort (*n* = 246)	Total (*n* = 1825)	*p*
Response				
pCR	350 (22.2)	46 (18.7)	396 (21.7)	0.220
Non‐pCR	1229 (77.8)	200 (81.3)	1429 (78.3)	
Sex				
Female	781 (49.5)	89 (36.2)	870 (47.7)	<0.001
Male	798 (50.5)	157 (63.8)	955 (52.3)	
Age (years)				
≤50	460 (29.1)	70 (28.5)	530 (29.0)	0.828
>50	1119 (70.9)	176 (71.5)	1295 (71.0)	
Pre‐CRT CA19‐9 (U/mL)	17.766 ± 15.72	20.52 ± 16.95	18.04 ± 15.92	0.014
Pre‐CRT CEA (ng/mL)	6.57 ± 6.15	6.45 ± 6.17	6.55 ± 6.15	0.771
Histopathology				
Adenocarcinoma	221 (14.0)	40 (16.3)	261 (14.3)	0.345
Signet‐ring cell carcinoma/ Mucinous adenocarcinoma	1358 (86.0)	206 (83.7)	1564 (85.7)	
Pre‐CRT T stage				
cT1	10 (0.6)	2 (0.8)	12 (0.7)	0.103
cT2	101 (6.4)	10 (4.1)	111 (6.1)	
cT3	662 (41.9)	90 (36.6)	752 (41.2)	
cT4	806 (51.0)	144 (58.5)	950 (52.1)	
Pre‐CRT N stage				
cN0	327 (20.7)	46 (18.7)	373 (20.4)	<0.001
cN1	820 (51.9)	173 (70.3)	993 (54.4)	
cN2	432 (27.4)	27 (11.0)	459 (25.2)	
Pre‐CRT MRI CRM				
Negative	616 (39.0)	118 (48.0)	734 (40.2)	0.008
Positive	963 (61.0)	128 (52.0)		
Pre‐CRT MRI EMVI				
Negative	449 (28.4)	80 (32.5)	529 (29.0)	0.189
Positive	1130 (71.6)	166 (67.5)	1296 (71.0)	
Pre‐CRT MRI tumor diameter (cm)	4.06 ± 2.35	4.26 ± 2.18	4.09 ± 2.33	0.187
Distance to the anal verge				
≤5 cm	661 (41.9)	98 (39.8)	759 (41.6)	0.001
5–10 cm	798 (50.0)	112 (45.5)	910 (49.9)	
≥10 cm	120 (7.6)	36 (14.6)	156 (8.5)	
Total neoadjuvant therapy				
No	1515 (95.9)	234 (95.1)	1749 (95.8)	0.547
Yes	64 (4.1)	12 (4.9)	76 (4.2)	
Interval to surgery (weeks)	10.07 ± 3.25	10.39 ± 3.08	10.11 ± 3.23	0.154
Radiation therapy courses				
Long course	1478 (93.6)	230 (93.5)	1708 (93.6)	0.949
Short course	101 (6.4)	16 (6.5)	117 (6.4)	

Abbreviations: CA19‐9, carbohydrate antigen19‐9; CEA, circumferential resection margin; CRM, circumferential resection margin; CRT, chemoradiotherapy; EMVI, extra‐mural venous invasion; LN, lymph nodes; MRI, magnetic resonance imaging; pCR, pathologic complete response.

Recent results from the training cohort showed significant differences for general information on patients who obtained pCR, including CEA, histopathology, Pre‐CRT T stage, N stage, TNM stage, TNT, and interval to surgery (All *p* < 0.05) (Table 2). Nevertheless, more stomas were avoided in pCR patients as seen in surgical treatment (73.9% vs. 69.7%, *p* = 0.043) (Table 3).

**TABLE 2:** Patient characteristics of pCR versus Non‐pCR in training cohort.

Variables	Non‐pCR (*n* = 1229)	pCR (*n* = 350)	Total (n = 1579)	*p*
Sex				
Female	607 (49.4)	174 (49.7)	781 (49.5)	0.915
Male	622 (50.6)	176 (50.3)	798 (50.5)	
Age (years)				
≤50	354 (28.8)	106 (30.3)	460 (29.1)	0.590
>50	875 (71.2)	244 (69.7)	1119 (70.9)	
Pre‐CRT CA19‐9 (U/mL)	17.93 ± 16.09	16.73 ± 14.37	17.66 ± 15.73	0.181
Pre‐CRT CEA (ng/mL)	7.03 ± 6.39	4.94 ± 4.91	6.57 ± 6.15	<0.001
Histopathology				
Adenocarcinoma	1024 (83.3)	334 (95.4)	1358 (86.0)	<0.001
Signet‐ring cell carcinoma/ Mucinous adenocarcinoma	205 (16.7)	16 (4.6)	221 (14.0)	
Pre‐CRT T stage				
cT1	7 (0.6)	3 (0.9)	10 (0.6)	<0.001
cT2	71 (5.8)	30 (8.6)	101 (6.4)	
cT3	463 (37.7)	199 (56.9)	662 (41.9)	
cT4	688 (56.0)	118 (33.7)	806 (51.0)	
Pre‐CRT N stage				
cN0	222 (18.1)	105 (30.0)	327 (20.7)	<0.001
cN1	656 (53.4)	164 (46.9)	820 (51.9)	
cN2	351 (28.6)	81 (23.1)	432 (27.4)	
Pre‐CRT TNM stage				
cII	222 (18.1)	105 (30.0)	327 (20.7)	<0.001
cIII	1007 (81.9)	245 (70.0)	1252 (79.3)	
Pre‐CRT MRI CRM				
Negative	435 (35.4)	181 (51.7)	616 (39.0)	<0.001
Positive	794 (64.6)	169 (48.3)	963 (61.0)	
Pre‐CRT MRI EMVI				
Negative	272 (22.1)	177 (50.6)	449 (28.4)	<0.001
Positive	957 (77.9)	173 (49.4)	1130 (71.6)	
Pre‐CRT MRI tumor diameter (cm)	4.03 ± 1.87	4.20 ± 3.55	4.06 ± 2.35	0.381
Distance to the anal verge				
≤5 cm	509 (41.4)	152 (43.4)	661 (41.9)	0.089
5–10 cm	617 (50.2)	181 (51.7)	798 (50.5)	
≥10 cm	103 (8.4)	17 (4.9)	120 (7.6)	
Total neoadjuvant therapy				
No	1189 (96.7)	326 (93.1)	1515 (95.9)	0.003
Yes	40 (3.3)	24 (6.9)	64 (4.1)	
Interval to surgery (weeks)	9.90 ± 3.09	10.66 ± 3.72	10.07 ± 3.23	0.001
Radiation Therapy Courses				
Long course	1146 (93.2)	332 (94.9)	1478 (93.6)	0.277
Short course	83 (6.8)	18 (5.10)	101 (6.4)	

Abbreviations: CA19‐9, carbohydrate antigen19‐9; CEA, circumferential resection margin; CRM, circumferential resection margin; CRT, chemoradiotherapy; EMVI, extra‐mural venous invasion; LN, lymph nodes; MRI, magnetic resonance imaging; pCR, pathologic complete response.

**TABLE 3:** Surgical treatment of pCR versus non‐pCR in the training cohort.

Variables	Non‐pCR (*n* = 350)	pCR (*n* = 1229)	Total (*n* = 1579)	P
Preservation of anal organs				
No	102 (8.3)	24 (6.9)	126 (8.0)	0.380
Yes	1127 (91.7)	326 (91.1)	1453 (92.0)	
Ostomy				
Yes	785 (63.9)	244 (69.7)	1029 (65.2)	0.043
No	444 (36.1)	106 (30.3)	550 (34.8)	
ypTNM stage				
yp 0	0 (0.0)	350 (100.0)	350	<0.001
yp I	404	0 (0.0)	404	
yp II	432	0 (0.0)	432	
yp III	393	0 (0.0)	393	

Abbreviation: pCR, pathologic complete response.

**FIGURE 2:**
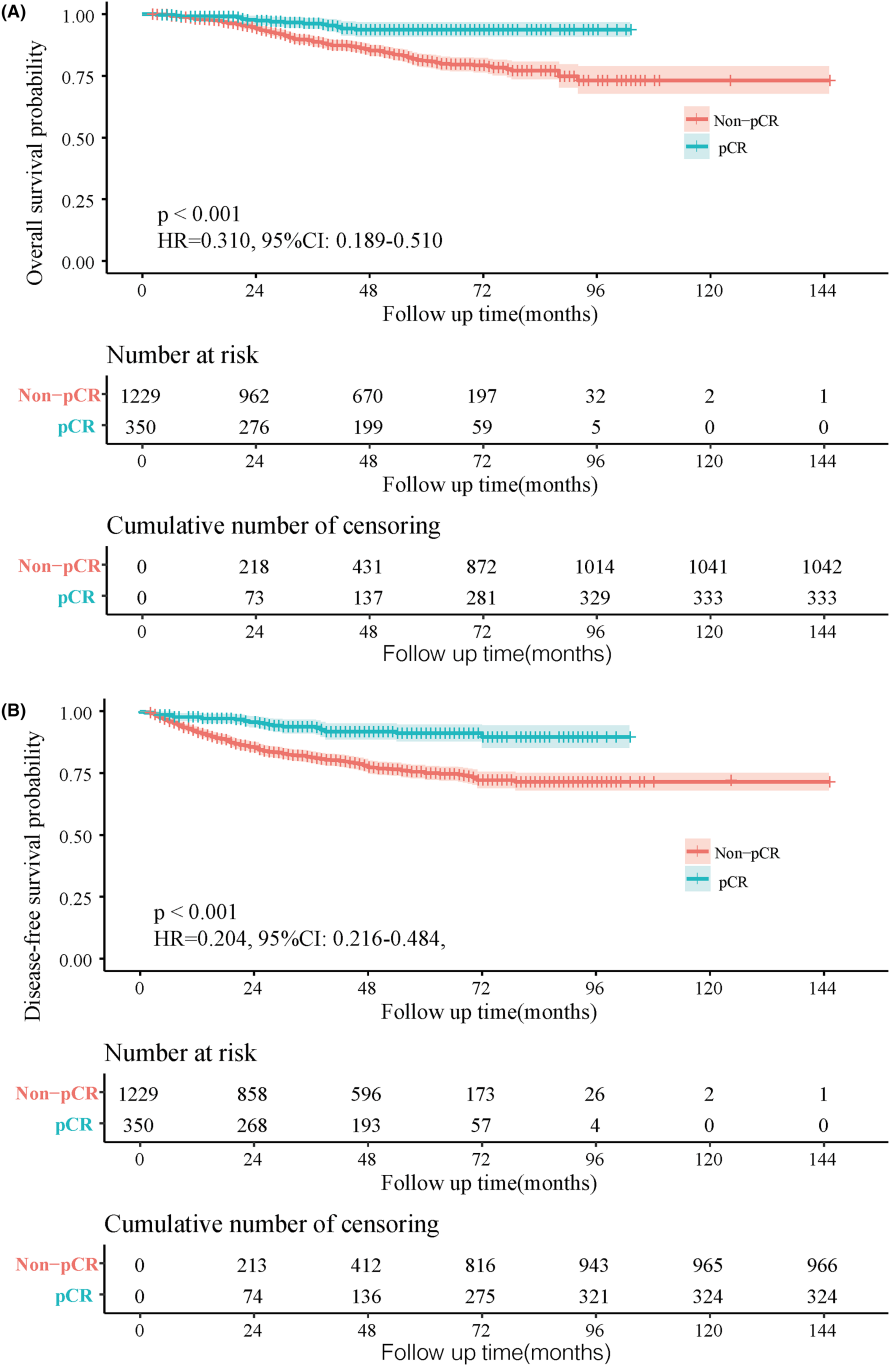
Kaplan–Meier curves of Overall survival (A), Disease‐free survival (B) between pCR and Non‐pCR groups in patients with locally advanced rectal cancer receiving neoadjuvant chemoradiotherapy.

After a median follow‐up time of 47.7 months (IQR 2–145) for the training cohort, shown as the survival curve results, OS (5‐year: 93.7% vs. 81.0%, HR = 0.310, 95%CI: 0.189–0.510, *p* < 0.001) and DFS (5‐year: 91.2% vs. 75.0%, HR = 0.204, 95%CI: 0.216–0.484, *p* < 0.001) were significantly better among patients with pCR than Non‐pCR (Figure 2A,B).

### 3.2 Independent predictors for pCR

Following univariable analysis, the multivariable Logistic analysis pCR was significantly associated with Pre‐CRT CEA (HR = 0.944, 95%CI: 0.921–0.968; *p* < 0.001), histopathology (HR = 4.608, 95%CI: 2.625–8.089; *p* < 0.001), Pre‐CRT T stage (HR = 0.793, 95%CI: 0.634–0.993; *p* = 0.043), Pre‐CRT N stage (HR = 0.727, 95%CI: 0.606–0.873; *p* = 0.001), Pre‐CRT MRI extra‐mural venous invasion (EMVI) (HR = 0.352, 95%CI: 0.262–0.473; *p* < 0.001), TNT (HR = 2.264, 95%CI: 1.280–4.004; *p* = 0.005) (Table 4).

**TABLE 4:** Univariate and multivariate Logistic regression analysis associated with pCR in the training cohort (*n* = 1579).

	Univariate analysis HR (95%CI)	*p*	Multivariate analysis HR (95%CI)	*p*
Sex (male vs. female)	0.916 (0.706–1.189)	0.510		
Age (≤50 years vs. >50 years)	0.784 (0.590–1.042)	0.093		
Pre‐CRT CA19‐9 (U/ml)	1.008 (0.999–1.018)	0.067		
Pre‐CRT CEA (ng/ml)	0.937 (0.913–0.962)	<0.001	0.944 (0.921–0.968)	<0.001
Histopathology (adenocarcinoma vs. signet‐ring cell carcinoma/Mucinous adenocarcinoma)	4.927 (2.756–8.809)	<0.001	4.608 (2.625–8.089)	<0.001
Pre‐CRT T stage (cT4, cT3, cT2 vs. cT1)	0.729 (0.566–0.940)	0.015	0.793 (0.634–0.993)	0.043
Pre‐CRT N stage (cN2, cN1, vs. N0)	0.728 (0.603–0.878)	0.001	0.727 (0.606–0.873)	0.001
Pre‐CRT MRI CRM (positive vs. negative)	1.213 (0.868–1.693)	0.258		
Pre‐CRT MRI EMVI (positive vs. negative)	0.333 (0.242–0.459)	<0.001	0.352 (0.262–0.473)	<0.001
Pre‐CRT MRI tumor diameter (cm)	1.026 (0.977–1.077)	0.310		
Distance to the anal verge (≥10 cm, 5–10 cm vs. ≤5 cm)	1.061 (0.854–1.318)	0.591		
Total neoadjuvant therapy (yes vs. no)	1.986 (1.070–3.686)	0.030	2.264 (1.280–4.004)	0.005
Interval to surgery (weeks)	1.035 (0.992–1.080)	0.110		
Radiation Therapy Courses (short vs. long)	0.861 (0.489–1.516)	0.605		

Abbreviations: CA19‐9, carbohydrate antigen19‐9; CEA, circumferential resection margin; CRM, circumferential resection margin; CRT, chemoradiotherapy; EMVI, extra‐mural venous invasion; LN, lymph nodes; MRI, magnetic resonance imaging; pCR, pathologic complete response.

### 3.3 Nomogram for pCR

**FIGURE 3:**
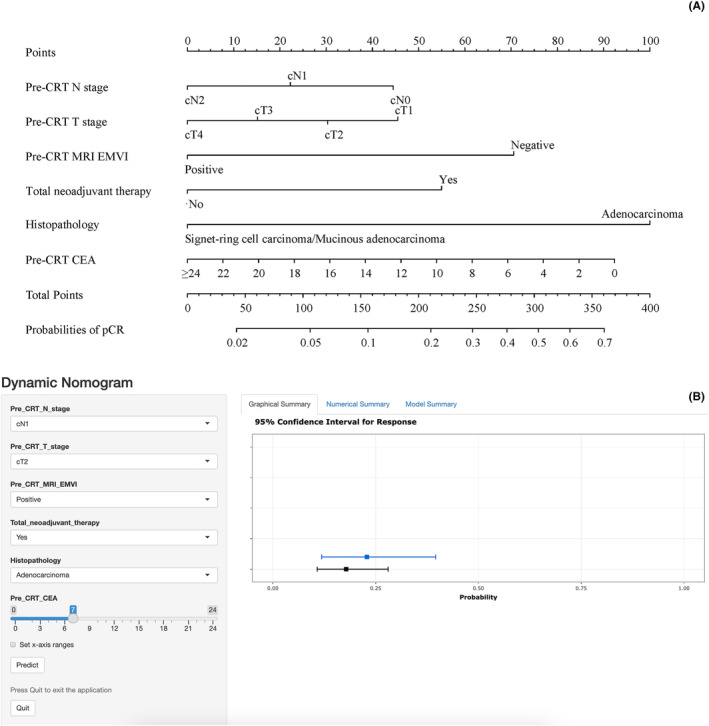
Nomogram (A) and online dynamic nomogram (B) to predict the probability of pCR in locally advanced rectal cancer after neoadjuvant chemoradiotherapy. The online dynamic nomogram was available at https://pcrpredict.shinyapps.io/LARC2/

A web‐based dynamic nomogram was established in the training cohort to predict the probability of pCR based on the results of multivariate Logistic regression analysis (Figure 3A). Through a browser, the online version of the nomogram can be logged in at https://pcrpredict.shinyapps.io/LARC2/. It is easy to determine the predicted probability of pCR by entering the patient's characteristics and reading the output calculated by the web server (Figure 3B).

Final formula for the nomogram = 22.0 × Pre‐CRT N stage (N0 = 2, N1 = 1, N2 = 0) + 15.0 × Pre‐CRT T stage (T1 = 3, T2 = 2, T3 = 1, T4 = 0) + 70.0 × Pre‐CRT MRI_EMVI (Negative = 1, Positive = 0) + 55.0 × Total neoadjuvant therapy (Yes = 1, No = 0) + 100 × Histopathology (Adenocarcinoma = 1, Signet‐ring cell carcinoma/Mucinous adenocarcinoma = 0) + 3.85 × Pre‐CR CEA (≥24 = 0).

The model predicted accuracy with a C‐index of 0.73 (95% CI: 0.70–0.75), with an average C‐index of 0.73 for the internal cross validation within the training cohort and 0.78 (95% CI: 0.72–0.83) for the external validation cohort, showing excellent model accuracy.

Delong test results showed that the model was statistically significantly different from the AUC of each variable (all *p* < 0.05), indicating that the model developed has an important gain value for clinical characteristics to predict pCR in rectal cancer (Table 5).

**TABLE 5:** The ROC curve analysis of predicted efficacy in the training and validation cohorts.

Predictive indicators	Training cohortn				Validation cohort			
	AUC (95%CI)	Sensitivity	Specificity	*p*‐value	AUC (95%CI)	Sensitivity	Specificity	*p*‐value
Nomogram	0.73 (0.70–0.75)	0.60	0.76	—	0.78 (0.72–0.83)	0.48	0.97	—
TNT	0.52 (0.49–0.54)	0.07	0.97	<0.001	0.56 (0.50–0.63)	0.15	0.98	<0.001
Histopathology	0.56 (0.54–0.59)	0.95	0.17	<0.001	0.55 (0.48–0.61)	0.91	0.18	<0.001
Pre‐CRT CEA	0.61 (0.59–0.63)	0.61	0.56	<0.001	0.56 (0.50–0.62)	0.99	0.25	<0.001
Pre‐CRT N stage	0.57 (0.54–0.59)	0.30	0.82	<0.001	0.55 (0.49–0.62)	0.30	0.84	<0.001
Pre‐CRT T stage	0.61 (0.59–0.64)	0.66	0.56	<0.001	0.65 (0.59–0.71)	0.67	0.65	0.003
Pre‐CRT MRI EMVI	0.64 (0.62–0.67)	0.51	0.78	<0.001	0.69 (0.63–0.75)	0.63	0.75	0.002

*Note*: *p*‐value represents the comparison of each indicator with Nomogram.

Abbreviation: AUC, area under the curve.

**FIGURE 4:**
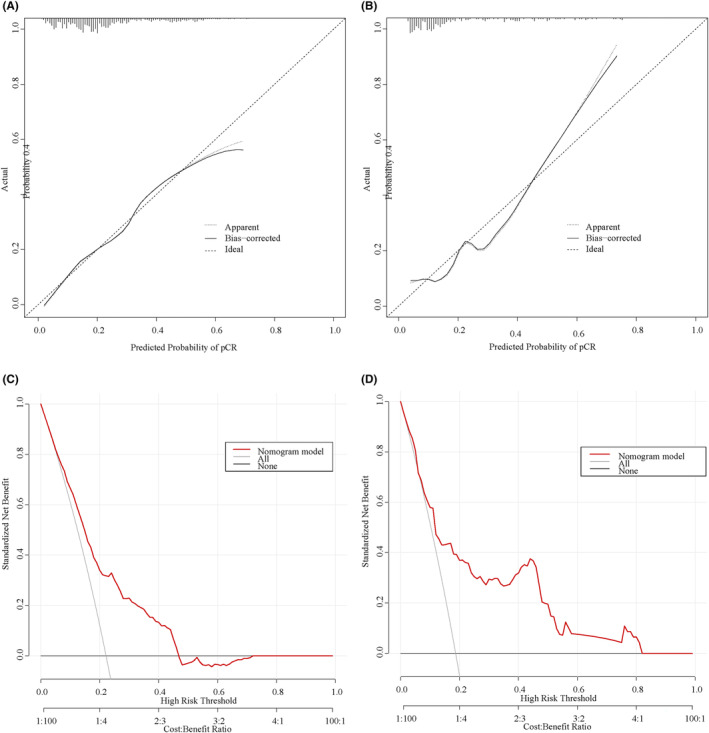
Calibration curves (A, B), and decision curve analysis (C, D) curves in the training and validation cohorts, respectively.

There is good agreement among the predicted probabilities of the predictive model and the actual observations in the training and validation cohorts, as shown by the calibration curves (Figure 4A,B). The result of the Hosmer‐Lemeshow‐test in the training cohort suggests a good fit for the model (*χ* = 6.087, *p* = 0.638). Decision curve analyses (Figure 4C,D) lie above the “none” and “all” lines, providing a quantitative indication of the clinical utility of the model.

## 4 DISCUSSION

To assure the reliability of the findings, further improve the accuracy of the predictive results, and address the lack of previous evidence, this retrospective project was conducted jointly at two large colorectal cancer centers. 1825 patients were included in this study and showed that there was an independent correlation between pCR and Pre‐CRT CEA, histopathology, Pre‐CRT T/N stage, Pre‐CRT MRI EMVI, and TNT. Meanwhile, to facilitate the application of the model to routine examinations, we publicize the online version of the nomogram established in this study on an open‐access website (URL: https://pcrpredict.shinyapps.io/LARC2/).

TNT was associated with improved delivery of systemic therapy and increased pCR to treatment, with pCR rates ranging from 17.2% to 38.5%, and it provides a promising platform for “watch‐and‐wait” protocol.^7^, ^11^ A large‐scale regression of 811 patients, 308 of whom were treated with TNT, further confirmed a significant association between TNT and pCR at 12 months (HR = 2.06; 95% CI, 1.44–2.96; *p* < 0.001).^7^ Similar to previous studies, although 64 only patients received TNT in the training cohort in this study, the pCR rate was high at 37.5% (24/64) in this subgroup, with multifactorial regression confirming a significant effect on pCR (HR = 2.264; 95% CI, 1.280–4.004; *p* < 0.001). However, there are still conflicting reports on whether TNT improves long‐term prognosis, and the long‐term effects of disease recurrence and OS need to be explored in future studies.^7^, ^12^, ^13^, ^14^

MRI is widely used in the diagnosis of rectal cancer due to its characteristics of having no radiation, high soft tissue resolution, and the ability to clearly identify the structure of each layer of the patient's rectal wall.^15^, ^16^ Pre‐CRT T/N stage is especially critical as an important basis for therapeutic decision‐making, and again different staging may have different outcomes on treatment response.^17^ One study result concluded that the cTNM stage was the only significant independent influence on pCR (HR = 6.92, 95% CI: 1.13–42.22, *p* = 0.038).^18^ However, the present study did not include cTNM in the model because of the statistical covariance between the cTNM stage and the T/N stage. In the future, we hope that our team can also conduct a multicenter prospective study based on this to develop a model with higher predictive accuracy for clinical use.

Similarly, the evaluation of tumor response after nCRT by MRI is particularly important.^19^ A study based on multiparametric MRI was performed to predict the probability of achieving pCR, providing important guidance for the adoption of “watch‐and‐wait” strategies with a good degree of predictive accuracy (AUC = 0.946).^20^ In previous studies, the author's team also incorporated MRI features for predicting good response after nCRT treatment, providing a prerequisite for determining whether LARC patients should receive nCRT.^21^ However, unlike previous studies, the present study focused on pCR as the endpoint, excluded some patients with distant metastases after nCRT, and constructed a web‐based dynamic nomogram that can be accessed on a public URL and is more convenient to use, which compensates for the previous shortcomings.

Previously, numerous studies have investigated the relationship between clinical variables and pCR, which also included independent influences similar to those reported in this study, such as low CEA levels^22^ and histopathology.^5^ In a study that included 260 rectal cancers, higher CEA levels (CEA >5 ng/dL) prior to nCRT were also considered to be associated with an incomplete tumor response.^23^ In addition, adenocarcinoma is often considered the better pathologic type and is most common in clinical practice.^5^ In the present study, adenocarcinoma accounted for a significantly higher proportion in the pCR group compared with non‐pCR (95.4% vs. 83.3%, *p* < 0.001). Mucinous adenocarcinoma has been reported to be a distinct pathologic entity with a poor prognosis, accounting for 5%–10% of rectal cancers^24^; and signet‐ring cell carcinoma is seen in only 0.1%–2.6% of colorectal cancer patients,^25^ both of which correlate with poor response to nCRT. Some researchers have attempted to attribute this poor response to nCRT to resistance to neoadjuvant chemotherapy in mucinous or signet‐ring cell carcinoma.^26^

In recent years, liquid biopsies including messenger ribonucleic acid (mRNA)^27^ and circulating tumor DNA (ctDNA)^28^ have also emerged as a hot topic in clinical research and have been applied to predict neoadjuvant efficacy. The probability of detecting ctDNA in LARC patients before undergoing nCRT has been reported to be as high as 77%^29^ and was significantly associated with shorter non‐metastasis‐free survival (HR = 3.8, 95% CI:1.2–11.7, *p* = 0.02).^30^ However, the relatively high price compared to commonly used indicators such as CEA and the small sample size of related studies have also limited the large‐scale application in clinics, including our center.^29^ Nevertheless, among the available studies, there are fewer findings regarding mRNAs that are differentially expressed in LARC after treatment with nCRT that could suggest different therapeutic responses, and thus as a basis for us to consider mRNAs as predictive biomarkers for neoadjuvant therapy is still significantly underdeveloped.^31^

Our study proved that patients with a non‐pCR to nCRT had worse survival prognoses and reduced stoma rates than those with a pCR, which was in line with the results of other LARC studies.^32^ Hence, early identification of possible acquired pCR after nCRT may help to develop personalized treatment strategies and predict prognosis, as well as to reduce unwanted side effects and economic burden. In previous studies, several models have been proposed to help predict pCR.^19^, ^33^, ^34^, ^35^ Ruud et al. indicated the potential value of PET/CT imaging in predicting the pCR response to LARC and developed a good‐performing online model (AUC = 0.78).^34^ However, PET/CT is relatively expensive and is not routinely used as a screening test for rectal cancer, therefore the utility of this model is still debatable.^1^ The development of radiomics in recent years has significantly improved the accuracy of predictive models. Cui et al. incorporated pretreatment multiparametric MRI features to construct a radiomics model to predict pCR, which improved the accuracy of the model to 0.966 (95% CI, 0.924–1.000).^19^ Notably, scanner limitations reduce the reproducibility of tumor features, image acquisition for radiomics distorts tissue properties, and radiomics requires specialized skills, limiting its routine application.^36^ To improve the utility, similar to this study, a nomogram (included 435 patients) was created by incorporating factors with good accessibility of relevant indicators (AUC = 0.81).^33^ However, these studies are mostly single‐center, retrospective studies with small sample sizes and a lack of external validation cohorts. Therefore, we attempted to overcome the above limitations by exploring the relationship between oncological features and pCR to further develop an early prediction model to guide the individualized treatment of patients in a better way. In addition, we provided an online version of the dynamic nomogram that is more easily accessible to physicians.

Our study has limitations. First, although this study was externally validated to have some external applicability, there is still some selection bias based on retrospective cohort analysis; excluding patients with incomplete clinical data, the probability of pCR may be underestimated or overestimated. Second, the radiotherapy dose of patients in this study was inconsistent, but it was shown that a short course of preoperative radiotherapy also resulted in good local control and excellent survival rates.^37^ Third, this study was limited to two large colorectal treatment centers in China, which is a regional limitation. Finally, patients' adherence to the diagnosis and treatment may also affect the results of pCR, therefore, when selecting patients at the beginning of our study, we excluded patients with incomplete data, patients who had not completed nCRT, etc. in order to minimize the effect of adherence on the results of treatment. In the future, with the deepening of colorectal cancer research, many new prognosis‐related variables will be gradually discovered, including BRAF, RAS gene status, and microsatellite status (MSS), and the nomogram model used for the prediction of pCR should be updated to obtain more accurate prediction assessment.

## 5 CONCLUSION

This study is a large retrospective cohort that demonstrated an independent correlation between Pre‐CRT CEA, histopathology, Pre‐CRT T/N stage, Pre‐CRT MRI EMVI, TNT, and the availability of pCR. pCR patients had a better prognosis including OS and DFS. A web‐based dynamic nomogram was successfully established for clinical use at any time.

## AUTHOR CONTRIBUTIONS

**Guancong Wang:** Conceptualization (equal); data curation (equal); formal analysis (equal); funding acquisition (equal); investigation (equal); methodology (equal); project administration (equal). **Jiasen Li:** Conceptualization (equal); data curation (equal); formal analysis (equal). **Ying Huang:** Methodology (equal). **Yincong Guo:** Methodology (equal).

## FUNDING INFORMATION

This study was supported by the Natural Science Foundation of Fujian Province (2023 J011819).

## CONFLICT OF INTEREST STATEMENT

The authors declare no competing interests.

## ETHICS STATEMENT

The Medical Ethics Committee of the Union Hospital of Fujian Medical University (Approval No. 2022KY134) and the Medical Ethics Committee of the Zhangzhou Affiliated Hospital of Fujian Medical University (Approval No. 2023LWB141) reviewed and agreed to waive informed consent this study. This study was conducted by relevant guidelines and regulations (Declaration of Helsinki).

## CONSENT FOR PUBLICATION

Not applicable.

## Data Availability

All data obtained or analyzed during this work are included within the article.
